# Supplementary material for: Kv10.1 Regulates Microtubule Dynamics during Mitosis
Source: Cancers (Basel). 2020 Aug 25;12(9):2409. doi: 10.3390/cancers12092409 (PMC7564071; doi:10.3390/cancers12092409)
Supplement: Supplementary file 1 [file cancers-12-02409-s001.zip › cancers-899677-original figrues and supplementary FigS1-S3/cancers-889677-suppl-final.docx]

Article

Kv10.1 Regulates Microtubule Dynamics during Mitosis

Naira Movsisyan and Luis A. Pardo





**Figure S1.** Kv10.1 downregulation activates DNA damage-induced checkpoint in the absence of DNA damage in HeLa cells. (**A**–**E**) Hela cells were transiently transfected with either *Scr-* or *KCNH1-* siRNA, synchronized with double thymidine block at the G1/S border, and subsequently released from the block. Samples were collected at the indicated time-points after release from the block and immunoblotted. Results of densitometry analysis of immunoblots are given (*n* = 3 independent experiments; mean ± SEM; two-way ANOVA, repeated measures, Bonferroni post hoc test; the exact *p*-values indicate the significance of the global difference between the groups).


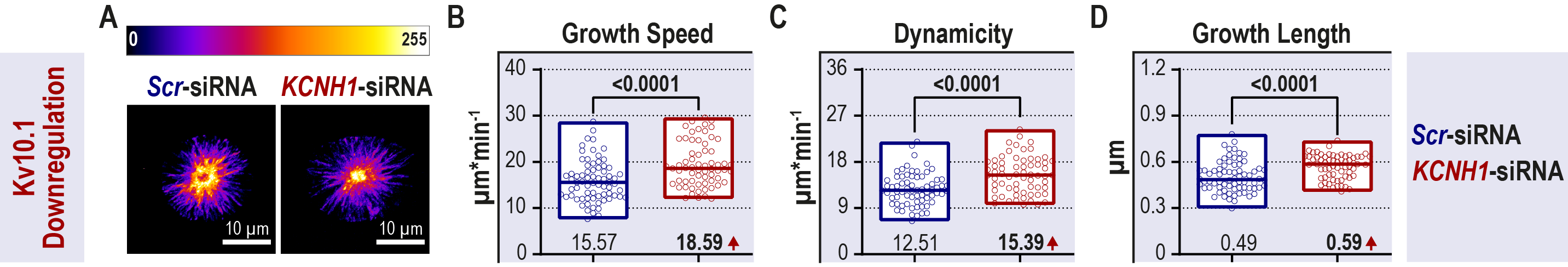


**Figure S2.** Kv10.1 downregulation leads to increased microtubule plus end growth rates in hTERT RPE1 cells. The cells were transiently co-transfected with the indicated siRNAs and pEB3-tdTomato and 48 h later treated with 2.5 µM DME for 4 h. Microtubule growth and shrinkage were tracked in a series of images taken every 500 ms for 1 min. (**A**) Maximal intensity projections of time-lapse images over the recorded time are shown (LUT is applied, scale bar, 10 μm). (**B**–**D**) Parameters calculated by U-Track software are plotted (*n* = 3 independent synchronization experiments; Mann-Whitney u-test, min to max range with median indicated as a line are shown).


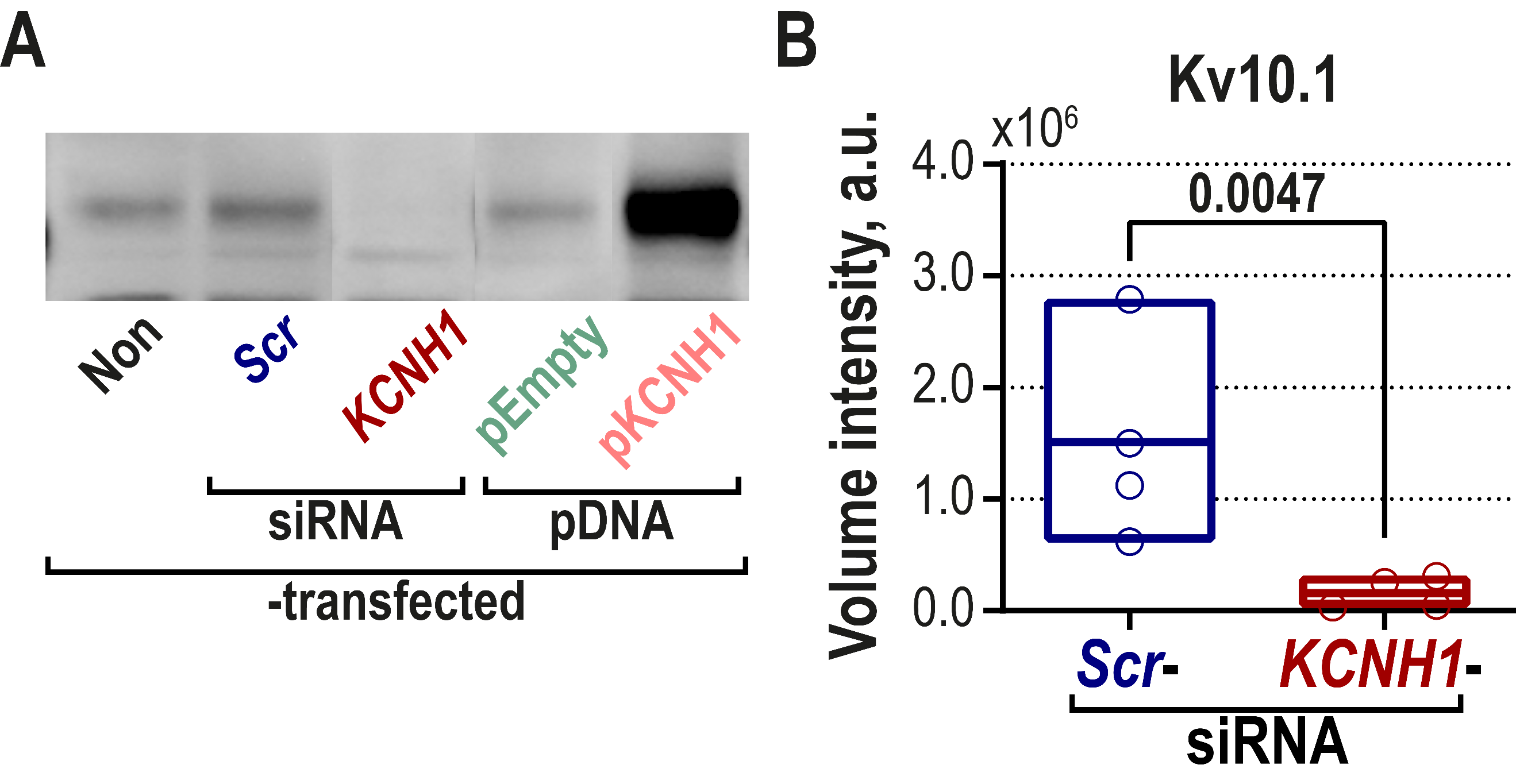


**Figure S3.** Efficacy of siRNA-mediated downregulation of Kv10.1 in HeLa cells. (**A**) The immunoblots show the expression level of Kv10.1 in unsynchronized cells 48 h after transfection. (**B**) Kv10.1 downregulation efficacy was quantified by densitometry analysis (*n* = 4 independent experiments; ratio paired t-test, min to max range with mean indicated as a line are shown).

| 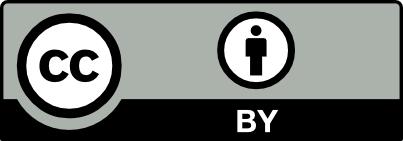 | © 2020 by the authors. Licensee MDPI, Basel, Switzerland. This article is an open access article distributed under the terms and conditions of the Creative Commons Attribution (CC BY) license (http://creativecommons.org/licenses/by/4.0/). |
| --- | --- |
