# Supplementary material for: Kv10.1 Regulates Microtubule Dynamics during Mitosis
Source: Cancers (Basel). 2020 Aug 25;12(9):2409. doi: 10.3390/cancers12092409 (PMC7564071; doi:10.3390/cancers12092409)
Supplement: Supplementary file 1 [file cancers-12-02409-s001.zip › cancers-899677-original figrues and supplementary FigS1-S3/cancers-889677-suppl-final.pdf]

cancers

MDPI

Article

Kv10.1 Regulates Microtubule Dynamics during Mitosis

Naira Movsisyan and Luis A. Pardo

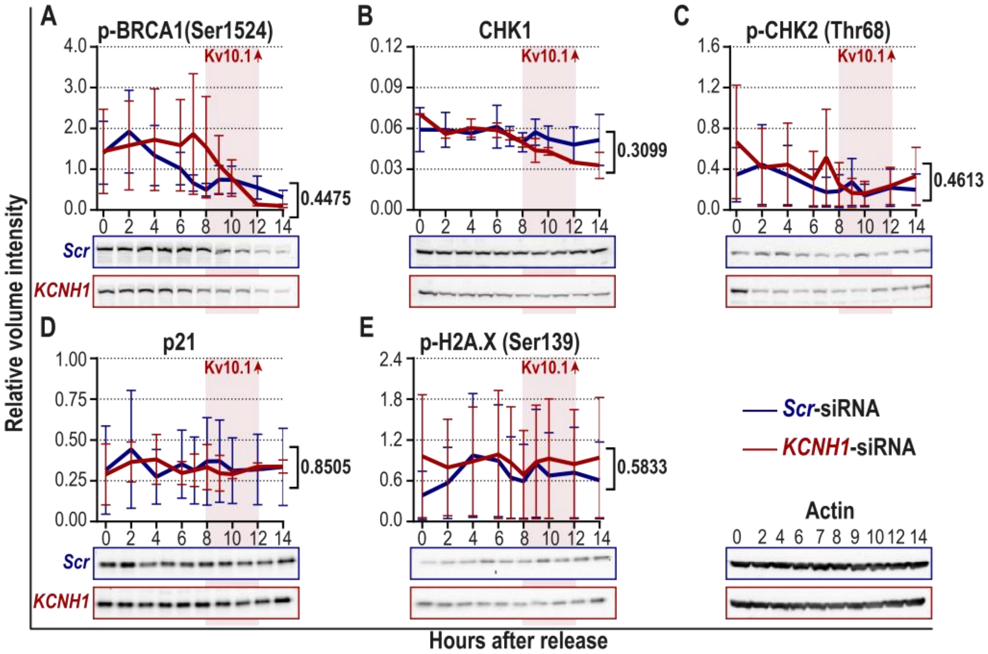

Figure S1. Kv10.1 downregulation activates DNA damage-induced checkpoint in the absence of DNA damage in HeLa cells. (**A**–**E**) Hela cells were transiently transfected with either *Scr*- or *KCNH1*- siRNA, synchronized with double thymidine block at the G1/S border, and subsequently released from the block. Samples were collected at the indicated time-points after release from the block and immunoblotted. Results of densitometry analysis of immunoblots are given (n = 3 independent experiments; mean ± SEM; two-way ANOVA, repeated measures, Bonferroni post hoc test; the exact p-values indicate the significance of the global difference between the groups).

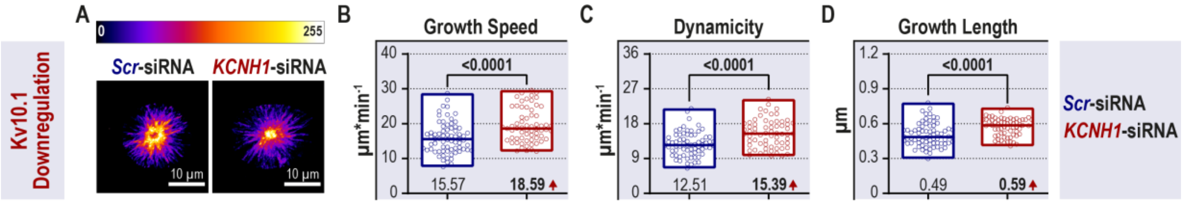

Figure S2. Kv10.1 downregulation leads to increased microtubule plus end growth rates in hTERT RPE1 cells. The cells were transiently co-transfected with the indicated siRNAs and pEB3-tdTomato and 48 h later treated with 2.5 µM DME for 4 h. Microtubule growth and shrinkage were tracked in a series of images taken every 500 ms for 1 min. (**A**) Maximal intensity projections of time-lapse images over the recorded time are shown (LUT is applied, scale bar, 10 µm). (**B**–**D**) Parameters calculated by U-Track software are plotted (n = 3 independent synchronization experiments; Mann-Whitney u-test, min to max range with median indicated as a line are shown).

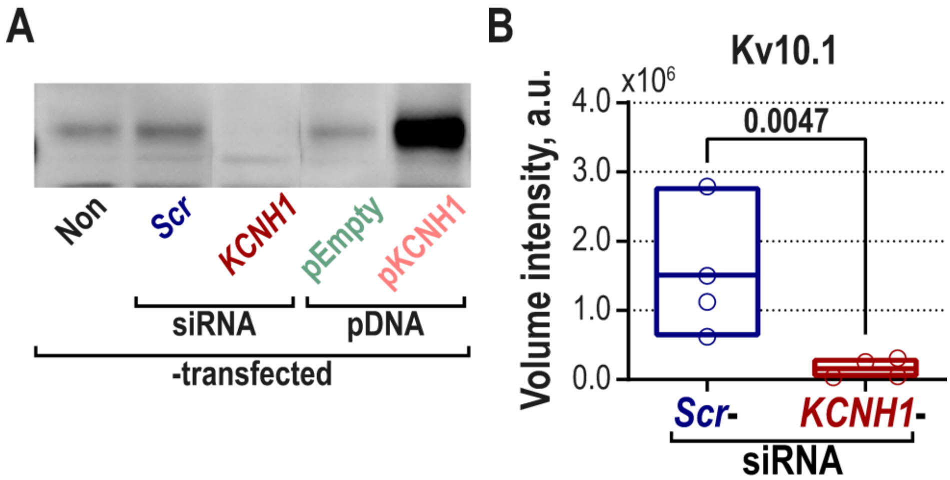

Figure S3. Efficacy of siRNA-mediated downregulation of Kv10.1 in HeLa cells. (**A**) The immunoblots show the expression level of Kv10.1 in unsynchronized cells 48 h after transfection. (**B**) Kv10.1 downregulation efficacy was quantified by densitometry analysis (n = 4 independent experiments; ratio paired t-test, min to max range with mean indicated as a line are shown).

© 2020 by the authors. Licensee MDPI, Basel, Switzerland. This article is an open access article distributed under the terms and conditions of the Creative Commons Attribution (CC BY) license (http://creativecommons.org/licenses/by/4.0/).
